# Targeted Silencing of NRF2 by rituximab-conjugated nanoparticles increases the sensitivity of chronic lymphoblastic leukemia cells to Cyclophosphamide

**DOI:** 10.1186/s12964-023-01213-1

**Published:** 2023-08-01

**Authors:** Atefeh Khodakarami, Mahsa Afsari Kashani, Atefeh Nazer, Armin Mahmoudsalehi Kheshti, Bentolhoda Rashidi, Vahid Karpisheh, Ali Masjedi, Shiva Abolhasani, Sepideh Izadi, Rafieh Bagherifar, Seyyed Sina Hejazian, Hamed Mohammadi, AliAkbar Movassaghpour, Abbas Ali Hosseinpour Feizi, Mohammad Hojjat-Farsangi, Farhad Jadidi-Niaragh

**Affiliations:** 1 Immunology Research Center, Tabriz University of Medical Sciences, Tabriz, Iran; 2 Department of Immunology, Faculty of Medicine, Tabriz University of Medical Sciences, Tabriz, Iran; 3 Student Research Committee, Tabriz University of Medical Sciences, Tabriz, Iran; 4 Institute of Experimental Hematology, School of Medicine, Technical University of Munich, 81675 Munich, Germany; 5 Center for Translational Cancer Research (TranslaTUM), School of Medicine, Technical University of Munich, 81675 Munich, Germany; 6 Department of Pharmaceutics, Faculty of Pharmacy, Tabriz University of Medical Sciences, Tabriz, Iran; 7 Non-Communicable Diseases Research Center, Alborz University of Medical Sciences, Karaj, Iran; 8 Hematology and Oncology Research Center, Tabriz University of Medical Sciences, Tabriz, Iran; 9 Bioclinicum, Department of Oncology-Pathology, Karolinska Institute, Stockholm, Sweden; 10 Research Center for Integrative Medicine in Aging, Aging Research Institute, Tabriz University of Medical Sciences, Tabriz, Iran

**Keywords:** Chronic lymphocytic leukemia (CLL), NRF2, Cyclophosphamide, Rituximab, Chemo-resistance

## Abstract

**Background:**

Targeting influential factors in resistance to chemotherapy is one way to increase the effectiveness of chemotherapeutics. The nuclear factor erythroid 2-related factor 2 (Nrf2) pathway overexpresses in chronic lymphocytic leukemia (CLL) cells and appears to have a significant part in their survival and chemotherapy resistance. Here we produced novel nanoparticles (NPs) specific for CD20-expressing CLL cells with simultaneous anti-Nrf2 and cytotoxic properties.

**Methods:**

Chitosan lactate (CL) was used to produce the primary NPs which were then respectively loaded with rituximab (RTX), anti-Nrf2 Small interfering RNA (siRNAs) and Cyclophosphamide (CP) to prepare the final version of the NPs (NP-Nrf2_siRNA-CP). All interventions were done on both peripheral blood mononuclear cells (PBMCs) and bone marrow mononuclear cells (BMNCs).

**Results:**

NP-Nrf2_siRNA-CP had satisfying physicochemical properties, showed controlled anti-Nrf2 siRNA/CP release, and were efficiently transfected into CLL primary cells (both PBMCs and BMNCs). NP-Nrf2_siRNA-CP were significantly capable of cell apoptosis induction and proliferation prevention marked by respectively decreased and increased anti-apoptotic and pro-apoptotic factors. Furthermore, use of anti-Nrf2 siRNA was corresponding to elevated sensitivity of CLL cells to CP.

**Conclusion:**

Our findings imply that the combination therapy of malignant CLL cells with RTX, CP and anti-Nrf2 siRNA is a novel and efficient therapeutic strategy that was capable of destroying malignant cells. Furthermore, the use of NPs as a multiple drug delivery method showed fulfilling properties; however, the need for further future studies is undeniable.

Video Abstract

**Supplementary Information:**

The online version contains supplementary material available at 10.1186/s12964-023-01213-1.

## Background

Chronic lymphocytic leukemia (CLL) is the most common type of leukemia in western adults aged 70 years old [1, 2]. Patients usually have lymphadenopathy, splenomegaly, and liver enlargement due to the aggregation of malignant B cells in these organs [3]. Moreover, CLL cells also overexpress CD5, CD20, CD19, and CD23 [4–6]. The clinical outcome for CLL is rather heterogeneous; some patients survive for many years without any treatment and end up dying from unrelated diseases, while others are not as lucky and die within 2–3 years after diagnosis, due to drug resistance and complications from aggressive therapies [7, 8]. The leukemic cells involve intricate signaling pathways that induce drug resistance and relapse episodes [9]. As a result, the need for development of new therapeutic approaches with lower resistance and better tolerance by the patients that interfere with these signaling pathways is always there. Among the CLL cell survival related signaling pathways that are well studied, Phosphatidylinositol-3-kinase/ Protein kinase B (PKB), also known as Akt (PI3K/AKT), Nuclear factor kappa-light-chain-enhancer of activated B cells (NFκB), Nrf2, Mitogen-activated protein kinase/ extracellular signal-regulated kinase (MAPK/ERK), WNT, and Neurogenic locus notch homolog protein 1 (NOTCH1) can be mentioned [10, 11]. Furthermore, elevated level of anti-apoptotic factors including B-cell lymphoma 2 (Bcl-2) and Myeloid leukemia cell differentiation protein1 (Mcl-1) is already confirmed in CLL [12, 13].

The human protein NF-E2-related factor 2 (Nrf2) is another overexpressed element seen in various hematologic malignancies such as Chronic myeloid leukemia (CML), Acute myeloid leukemia (AML), Acute promyelocytic leukaemia (APML), Acute promyelocytic leukemia (APL), CLL, and Acute lymphoblastic leukemia (ALL) which is also seen in many solid malignancies like pulmonary, head and neck masses, adenocarcinoma, and large cell neuroendocrine carcinoma [14–16]. Nrf2 is a transcriptive regulator that causes expression of cytoprotective and oxidative stress-countering genes to sustain redox homeostasis [17]. After exposure to oxidative and electrophilic stresses in normal cells, Kelch like ECH associated protein 1 (Keap1) an Nrf2 inhibitor undergoes structural changes leading to Nrf2 release into the cytoplasm [18]. Then, the Nrf2 gets carried into the nucleus where another transcription factor called small MAF proteins is present and they make a heterodimer together. The complex is then bound to the antioxidant response element (ARE), an order of nucleotides in DNA chromosomal enhancer which induces expression of genes in charge of redox homeostasis to shield the cell against DNA damage [3, 19].

**Fig. 1:**
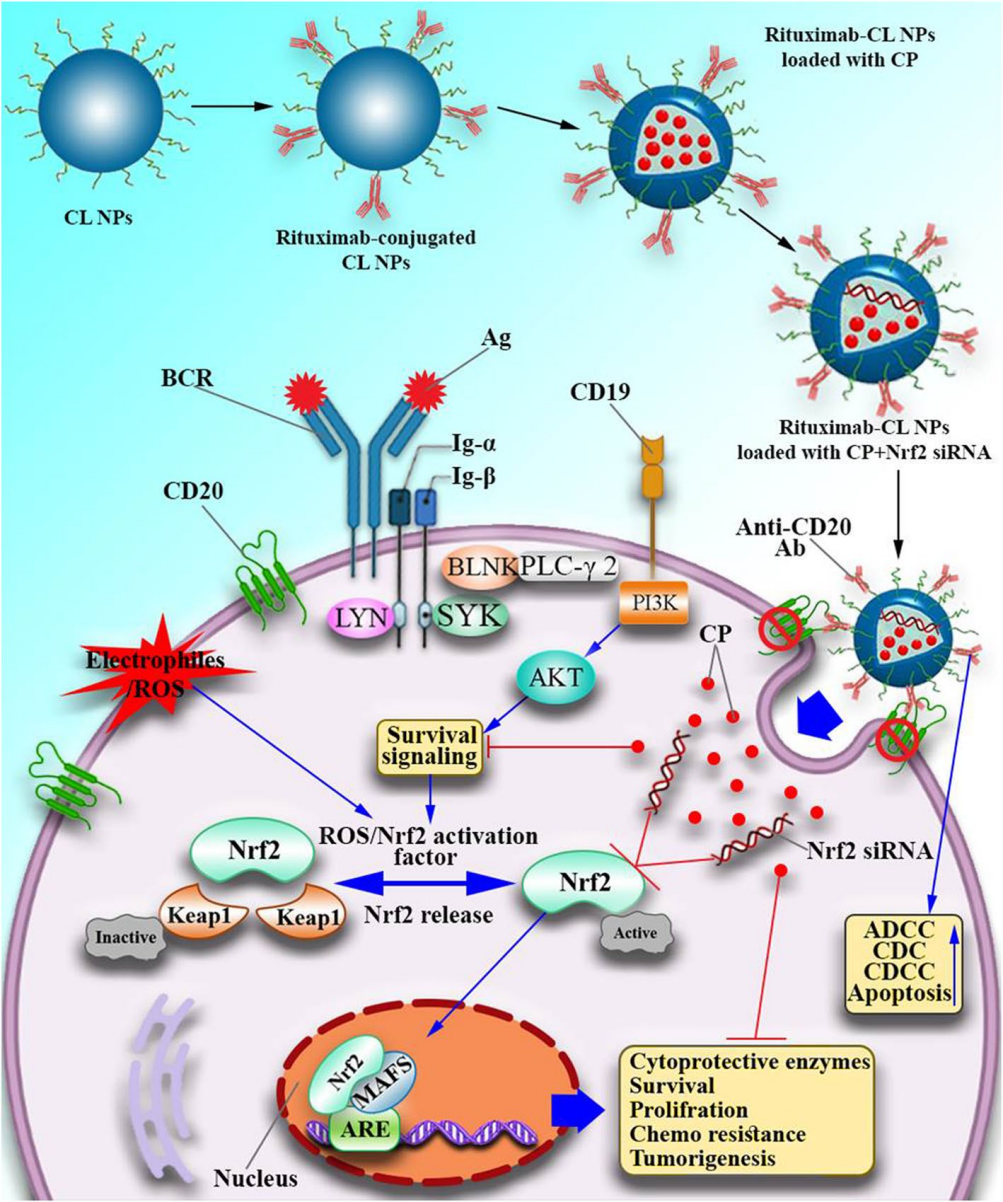
RTX-CL-NPs loaded with CP and anti-Nrf2 siRNA (NP-Nrf2_siRNA-CPs) induced pathways within CLL malignant cells. As pictured here, RTX causes specific targeting of leukemic CD20 + cells by NP-Nrf2_siRNA-CPs which enhances cell entry efficacy. Subsequently, Acidic microenvironment of malignant cells causes CP and anti-Nrf2 siRNA release from NPs. Anti-Nrf2 siRNA interferes with Nrf2 mediated cellular pathways which lead to apoptosis induction, decreased drug resistance, and suppressed cellular proliferation. Furthermore, RTX promotes cell apoptosis (PCD) making malignant CLL cells to be more susceptible to CP

Hyperactivation of Nrf2 is exploited by most common cancer cells to augment cell expansion and survival which is also corresponding to Bcl-2 and Mcl-1 hyperexpression [15]. Moreover, silencing Nrf2 signaling significantly increases the efficacy of conventional therapies such as chemotherapy and could be regarded as a favorable approach to enhance efficacy of these agents such as CP [20, 21] (Fig. 1). CP is used to treat various malignancies such as leukemia, lymphoma, ovarian adenocarcinoma, bladder, breast, and lung cancers, neuroblastoma, retinoblastoma, Ewing's sarcoma, and Wilms' tumor [22]. After binding to DNA molecules, CP alkylates DNA, leading to the formation of DNA crosslinks and thereby effectively preventing DNA replication [22]. However, the problem is that gradual reduction in patients’ sensitivity to CP is preventive of achieving best clinical results and needs more considerations [23]. Accordingly, inhibiting the expression of drug resistance-retaining molecules such as Nrf2 using small interfering RNA (siRNA) molecules has been demonstrated to be effective [24, 25]. However, targeted treatment with siRNA accompanies some drawbacks including low stability, effecting undesired targets, RNase and phosphatase induced vulnerabilities, and poor pharmacokinetics which all can make the treatment less effective [26]. To remove these barriers, delivery techniques such as nanocarriers have been used by several researchers [27, 28]. Transporting chemotherapeutic agents with nanoparticles (NPs) increases the drug solubility and decreases the minimum required therapeutic dose [29, 30].

The nonspecific nature of most therapeutic agents against malignant cells used in the treatment of hematopoietic malignancies is a problematic issue, and solving it is a matter of great importance [31]. Here, in this paper, knowing that leukemic cells have high expression of CD20 molecule, we used anti-CD20 antibody, Rituximab (RTX), to deliver therapeutic agents to CLL malignant cells. For this purpose, we attached RTX antibodies to Chitosan lactate nanoparticles loaded with anti-Nrf2 siRNA and CP to specifically target CLL cells ex vivo for the first time. Considering the increased CLL cells' sensitivity to CP and causing enhanced programmed cell death (PCD) or apoptosis, and suppressed malignant cell proliferation, our findings suggest a promising potential of using these NPs to achieve more efficient treatment of CLL patients.

## Methods

### Materials

CP was purchased from Cayman Chemical Company (IRC No: 7069750324959135). The Rituximab chimeric mAb (anti-CD20) was obtained from Sigma-Aldrich. N-hydroxysuccinamide (NHS), 1-Ethyl-3-(dimethylaminopropyl) carbodiimide hydrochloride (EDC) was supplied by Sinopharm Chemical Reagent Co., Ltd. (Shanghai, China). Human Nrf2 gene targeting siRNA (sense: 50-CAT CCA GTC AGA AAC CAG TGG-30 and antisense: 50-GCA GTC ATC AAA GTA CAA AGC AT-30) and negative control siRNA (NC-siRNA) were provided from Santa Cruz Biotechnology, Inc. Cell Proliferation Enzyme-Linked ImmunoAssay (ELISA) bromodeoxyuridine (BrdU) (colorimetric) from (Roche Molecular Biochemicals, Indianapolis, IN). The MTT Cell Proliferation Assay Kit (3-(4, 5-dimethylthiazol-2-yl)-2, 5-diphenyl tetrazolium bromide) was purchased from the American Type Culture Collection (ATCC® 30-1010 K) and exploited as directed by the manufacturer. The Annexin V-FITC Apoptotic Detection Kit was purchased from Sigma-Aldrich, Inc.

### Biological specimen

By following the principles of the Declaration of Helsinki, after getting permission from the ethics committee of Tabriz University of Medical Sciences (ethic code: IR.TBZMED.REC.1400.053), heparinized blood samples were taken from untreated CLL patients at the Shahid Ghazi Hospital of Tabriz. Afterwards, primary leukemia cells were obtained from peripheral blood and bone marrow of eleven patient samples with confirmed CLL using Ficoll Paque TM Plus (GE Healthcare, Uppsala, Sweden). The main features of the patients are given in the Supplementary Table S1. Both peripheral blood mononuclear cells (PBMCs) and bone marrow mononuclear cells (BMMCs) of the patients were studied. For this purpose, they were incubated in Roswell Park Memorial Institute medium (RPMI-1640) medium consisting of 20% fetal bovine serum (FBS) and 2% L-glutamine. Viable cells were enumerated in advance of the downstream analysis.

### Preparation of the nanoparticles

Similar to our prior studies [32, 33], we used Chitosan lactate (CL) based nanocomplexes in this study, however, with minor alterations. In summary, CL copolymer was synthesized by dissolving 200 mg low molecular weight (50 kDa) chitosan in 6.5 ml lactic acid and stirring it for 20 min. Then, 13.5 mL distilled water was added to the solution and blended for a more 12 h period. In the final step, the solution was lyophilized. For conjugating antibodies to CL, 100 μg/µl of RTX in association with a mixture of EDC and NHS (0.5 mg/ml) was mixed with 5 ml of CL dissolved in Phosphate buffered saline (PBS) (5 mg/ml) with neutral pH and incubated for 45 min at ambient condition under gentle stirring. Then, the reaction solution was centrifuged (6,000 rpm, 2 h), washed, and suspended in PBS to exclude free RTX antibodies. A Pierce™ BCA Protein Assay Kit was exploited to assess the conjugation efficiency of CL and RTX. In brief, the protein concentration was assessed by mixing bicinchoninic acid with supernatant (ratio of 1:1) and incubating at 37 °C for 2 h. A UV/VIS spectrophotometer (562 nm) was then used to measure the protein concentration, and the conjugation efficiency was determined by the following equation [34]:

1 $$\mathrm{Conjugation}\;\mathrm{efficiency}\left(\%\right)=\frac{\left(\mathrm{Initial}\;\mathrm{RTX}\right)-(\mathrm{free}\;\mathrm{RTX})}{\mathrm{Initial}\;\mathrm{RTX}}\times100$$

To load CP, a 20 mg/mL concentration of CP with a volume of 1 mL was added to 5 mL of RTX-conjugated CLs (from now on, by NP, we mean this structure) under gentle stirring at ambient conditions for 12 h. Isolation of CP-loaded NPs (NP-CP) was done by centrifugation (5,000 rpm, 1 h). Deposited NP-CP were washed and centrifuged again to remove excess free CP. The final form of NPs (NP-Nrf2_siRNA-CP) was achieved by adding 20 μL anti-Nrf2 siRNA (equivalent to 5 μg) to 2 ml NP-CP and vortexing (3000 rpm) for 1 h at ambient conditions. Moreover, to explore the bare effect of anti-Nrf2 siRNA on malignant CLL cells a specific form of NPs were provided which only contained anti-Nrf2 siRNA and not CP (NP-Nrf2_siRNA).

### Main features of the NP-Nrf2_siRNA-CP

#### Size and charge of NP-Nrf2_siRNA-CP

Photon correlation spectroscopy (PCS) (Nano-ZS; Malvern equipment, Malvern, UK) was used to assess the physical and chemical properties of various forms of produced NPs including scale, zeta potential, and polydispersity index (PDI). The wavelength, detection angle and temperature were respectively set to 630 nm, 90°, and 25° C during the process of all analysis.

#### Chemical structure and stability of NP-Nrf2_siRNA-CP

To explore the chemical structure of NP-Nrf2_siRNA-CP Fourier Transmission Infrared Spectroscopy (FTIR) was used (CARY; 610 models, USA). The stability of NP-Nrf2_siRNA-CP within serum was examined based on agarose gel electrophoresis at various periods. For this purpose, 500 µL of prepared NP-Nrf2_siRNA-CP (containing 5 µg siRNA) was mixed with 250 µL fetal bovine serum (FBS) and then incubated for 48 h with a temperature setting of 37° C. An aliquot of samples was taken at various periods (2, 4, 8, 16, 24, 36, and 48 h) and analyzed by agarose gel electrophoresis [32].

#### siRNA/drug release pattern

Similar to the procedure described in our earlier report [35], the in vitro release pattern of CP and anti-Nrf2 siRNA from NPs in diverse pH setting including 5.5 and 7.4 were evaluated using a NanoDrop spectrophotometer (Thermo Fisher Scientific: 2000c model, USA) [36]. For this purpose, the NP-Nrf2_siRNA-CPs were suspended in PBS (50 mL) at temperature of 37° C while being gently stirred with a magnetic stirrer inside a dialysis bag with a molecular weight limit of 15,000 Da (enabling siRNA and CP with molecular weights of 13.2 g/mol and 261.08 g/mol, respectively to pass through the dialysis bag). A proper amount of medium (2 mL) was then replaced with a similar proportion of fresh medium. The materials were ultimately evaluated using UV-2550 spectrophotometry.

#### Drug encapsulation efficiency

**Fig. 2:**
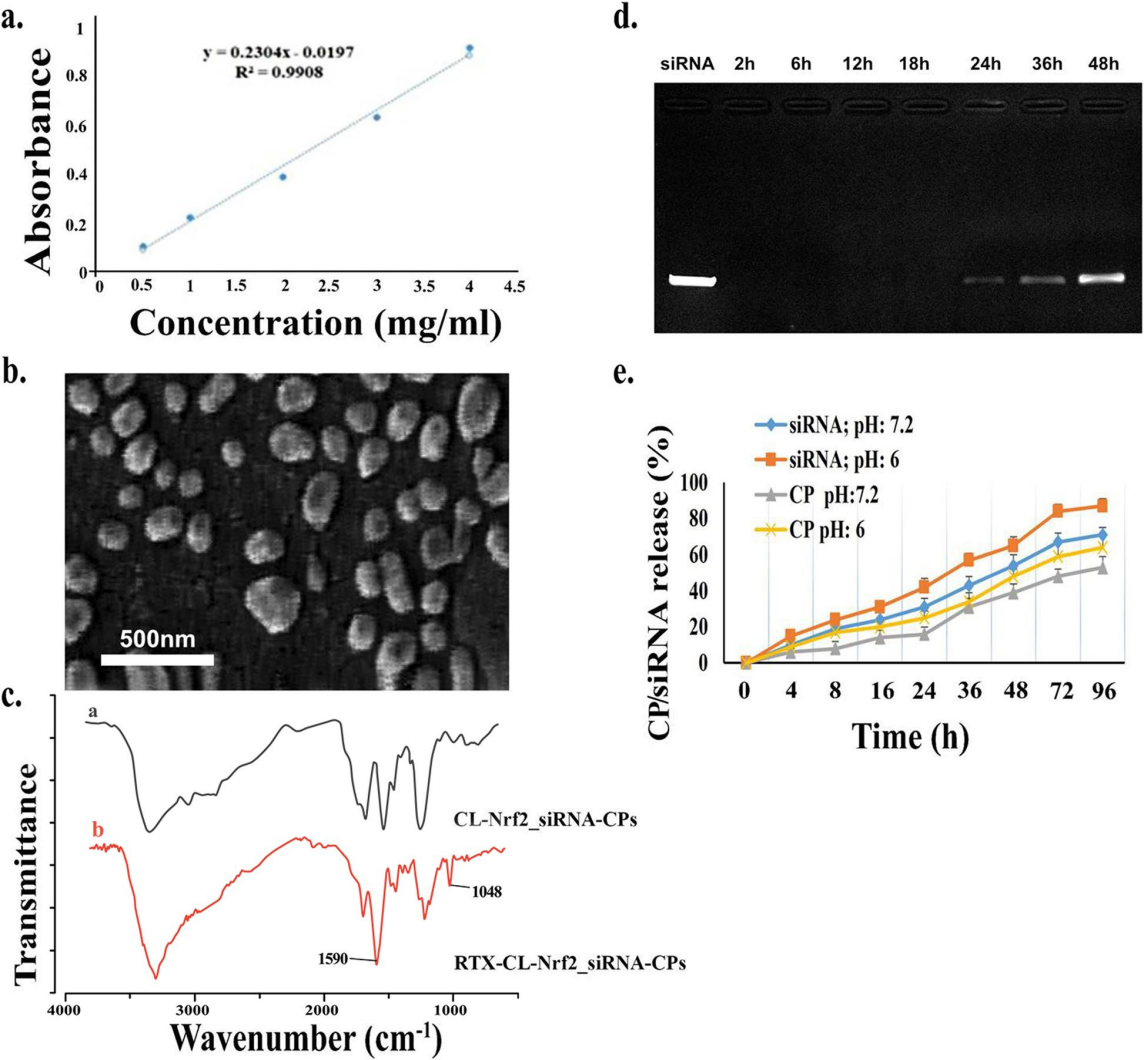
Characterization of NP-Nrf2_siRNA-CPs. (**a**) Calibration curve of CP in water, (**b**) Scanning Electron microscopy image of NP-Nrf2_siRNA-CPs, (**c**_a_) CL-Nrf2_siRNA-CPs chemical structure based on Fourier Transform Infrared spectroscopy analysis, (**c**_b_) FTIR results of RTX-CL-Nrf2_siRNA-CPs (**d**) Agarose gel electrophoresis of NP-Nrf2_siRNA-CPs confirming serum stability (lane 1: naked Nrf2 siRNA, 2: 4 h, 3: 6 h, 4: 8 h, 5: 10 h, 6: 12 h, 7: 14 h, 8: 16 h, 9: 18 h), (**e**) UV spectrophotometer analysis demonstrating in vitro release profile of NP-Nrf2_siRNA-CPs in neutral (PH = 7.2) and acidic (PH = 6) environments

We prepared a 4 mg/ml concentration of CP stock solution within water to evaluate the drug encapsulation efficiency. The solution was then scanned in the UV/Vis spectrophotometer in the 200–400 nm range, yielding a maximum wavelength (λ_max_) of 210 nm. To design a CP standard calibration curve in water (Fig. 2a), diluted version of the stock solution with different concentrations including 3, 2, 1, and 0.5 mg/ml were prepared. The UV spectroscopy of each solution was performed with water serving as a blank. A standard curve was created for the whole range of 0.5 to 4 mg/ml.

The amount of CP in NPs was determined using UV/Vis spectrophotometer to calculate the drug encapsulation efficiency. For this purpose, NP-CP-Nrf2_siRNAwere centrifuged at 12,000 rpm. The NP pellets and the supernatant were then separated. UV/Vis determined the absorbance of the supernatant at 210 nm, and the amount of CP in the solution (free or unencapsulated CP) was obtained using the calibration curve. Finally, the drug loading (DL%) and encapsulation efficiency (EE%) were computed based on the formula below [34]:

2 $$EE\%=\frac{\mathrm{initial}\;\mathrm{weight}\;\mathrm{of}\;\mathrm{the}\;\mathrm{drug}-\mathrm{weight}\;\mathrm{of}\;\mathrm{the}\;\mathrm{drug}\;\mathrm{in}\;\mathrm{the}\;\mathrm{supernatant}}{\mathrm{initial}\;\mathrm{weight}\;\mathrm{of}\;\mathrm{the}\;\mathrm{drug}}\times100$$

3 $$DL\%=\frac{\mathrm{amount}\;\mathrm{of}\;\mathrm{drug}\;\mathrm{in}\;\mathrm{NPs}}{\mathrm{amount}\;\mathrm{of}\;\mathrm{drug}-\mathrm{loaded}\;\mathrm{NPs}}\times100$$

#### Shape of NP-Nrf2_siRNA-CP

The shape of NP-Nrf2_siRNA-CP was examined using scanning electron microscopy (SEM) (HITACHI; H9500 model, Japan) [37]. In summary, a drop of NP-Nrf2_siRNA-CP containing solution was applied to the lam and allowed to dry before being placed in an argon atmosphere and covered with a carbon layer. We used Anix Emica software to evaluate the samples.

#### Cellular uptake of NP-Nrf2_siRNA-CP

To examine the cellular uptake, specific form of NP-Nrf2_siRNA-CP were provided using CY5-conjugated siRNA (NP-CP-Nrf2_siRNA-CY5). Then 6-well culture plates with a complete cell growth medium were used to culture 5 × 10^5^ CLL cells. The cells were incubated at 37 °C with 5% CO2 after being treated with NP-CP-Nrf2_siRNA-CY5s. After a full day, the cells were washed with PBS and then fixed with a fixation buffer consisting of PBS and formaldehyde for 30 min. The next step involved washing the cells twice and staining them for three minutes with the nuclear stain, DAPI (0.6 mg/ml) and then washing them again with PBS. Finally, transfected cells were evaluated using confocal microscopy (Nikon Eclipse Ti, Tokyo, Japan) to examine the transfection yield of NP-CP-Nrf2_siRNA-CY5s [38].

### Evaluation of cell proliferation

Cell Proliferation ELISA, BrdU (colorimetric) kit was used to analyze the cell proliferation (Merck, Darmstadt, Germany). In brief, 2 × 10^5^ CLL cells were cultured overnight in a 96-well plate and then exposed to different therapeutic agents based on our treatment groups (NP (RTX-containing NP): 20 µM, NP-Nrf2 siRNA: 9.63 µM, CP: 145.2 µM, NP-CP: 122.2 µM, NP-Nrf2 siRNA-CP: 120.2 µM). BrdU labeling solution (20 μl/well) was added to each well for 4 h before utilizing FixDenat solution (100 μl/well). The cells were then treated for 90 min with 100 μl Anti-BrdU-POD stock. After three washes, the wells were incubated for 30 min with TMB substrate (100 μl) at room temperature (RT) in the dark, the color intensity of wells was proportional to the amount of BrdU incorporated in the proliferating cells. Subsequently, 100 µL of Acid Stop Solution was used to stop the reaction of each well, resulting in change of positive well’s color from blue to bright yellow. Then Spectrophotometric a microplate reader (Bio-Rad, Hercules, CA, USA) set at a dual wavelength of 450/550 nm was used to count the BrdU-positive cells. The amount of OD of untreated cells was considered as a reference and the rest of the groups were normalized to it and expressed as a percentage.

### Quantitative reverse transcriptase-polymerase chain reaction (qRT-PCR)

After incubation of transfected cell for 48 h, the total RNA was extracted using Trizol reagent (Takara Bio Inc., Japan) based on the manufacturer’s instructions. RNA transcription to cDNA was performed with the help of One-Step SYBR® RT-PCR Kit (Takara Bio Inc., Japan). To do so, 2 µg of total RNA was used. Then, cDNA was mixed with SYBR® Green Real-time PCR master mixture (Takara Bio Inc., Japan) consisted of 10 μM forward primer (0.5 µl), 10 μM reverse primer (0.5 µl), 2X concentration of SYBR Premix Ex Taq (10 µl), and RNase-free water (7 μl). The final mixture was transferred to the Light-Cycler480 real-time PCR system (Roche) to perform the qRT-PCR. Before the main reaction which was conducted using 2 µl of cDNA, a test reaction was carried out using 1 ml of cDNA. The system setting included an initial temperature of 95 °C for 30 s to induce denaturation and activation. Afterwards, intermittent shift of temperature from 95° C for 5 s to 60° C for 30 s was done to allow amplification and quantification for 40 cycles. During the process, melting and standard curves were created to confirm specificity and used to determine the relative amount of desired gene mRNA. Analysis was performed using the delta delta CT method (ΔΔCT Method) based on β-actin mRNA level which is a housekeeping gene. Gene primer sequences are available in Table S2.

### Western blotting

After various combined treatments based on our treatment groups, the PBMC and BMMC samples were resuspended in the RIPA lysis buffer. Subsequently, using the BCA protein analysis kit (Thermo Fisher), the concentration of cell lysate suspensions was determined, and an equal quantity of protein and protein markers were separated on 12% Bis–Tris acrylamide gels (stacking gel/separator) and transferred to nitrocellulose membranes (PVDF). Membranes were blocked with 5% skimmed milk in TBS buffer (170 mm NaCl, 70 mM Tris, pH 7.7) for 1 h at room temperature or overnight at 4 °C. Afterward, membranes were incubated with monoclonal antibodies against the Nrf2 gene (1: 1000), bcl2 (1: 400), Bax (1: 1000), and polyclonal antibodies against β-actin (1: 2000) overnight. After three washes with PBST and 1 h of incubation with anti-rabbit IgG-HRP antibodies, the western blot detection kit (Thermo Fisher Scientific) was used to measure the relative amounts of targeted proteins.

### Evaluation of cytotoxicity and cell death

The MTT (3-(4, 5-dimethylthiazol-2-yl)-2, 5-diphenyl tetrazolium bromide) was used to determine the cell toxicity of several treatments. 2 × 10^5^ of patient-derived PBMCs and BMMCs were separately added to 96-well plates containing 200 µl complete culture medium and then incubated at 37° C under 5% CO2 for 24 h. Afterwards, plates were centrifuged (150 × g, 10 min), and the finest medium was replaced with 100 µl fresh medium which contained therapeutic agents (NP (RTX-containing NP): 20 µM, NP-Nrf2 siRNA: 9.63 µM, CP: 145.2 µM, NP-CP: 122.2 µM, NP-Nrf2 siRNA-CP: 120.2 µM). Then the plates were incubated for 24 h and this time, the medium was replaced with a mixture of 100 µl fresh medium and 10 µl MTT solution (final concentration; 0.5 mg/mL). The wells were incubated for 4 h at 37° C before removing the medium and replacing it with 100 µl of Dimethyl sulfoxide (DMSO) which improves formazan solubilization. Finally, a plate reading spectrophotometer (Synergy 4, Bio Tec, USA) was used to measure the absorbance of each well. The cell viability percent was estimated using the following formula [39]:

4 $$\mathrm{Viability}=\frac{(\mathrm{OD}\;\mathrm{treated}\;\mathrm{well}\;\lbrack-\mathrm{blank}\rbrack)}{(\mathrm{mean}\;\mathrm{OD}\;\mathrm{control}\;\mathrm{well}\;\lbrack-\mathrm{blank}\rbrack)}\times100$$

### Evaluation of cell apoptosis

To explore one of the principal goals of the study, effect of different therapeutic agents based on our treatment groups on apoptosis of CLL cells, 2 × 10^5^ of PBMCs and BMMCs from CLL patients were separately added to 96-well plates and incubated with different therapeutic agents (NP (RTX-containing NP): 20 µM, NP-Nrf2 siRNA: 9.63 µM, CP: 145.2 µM, NP-CP: 122.2 µM, NP-Nrf2 siRNA-CP: 120.2 µM). for over 24 h and then washed with PBS. Cells were then treated for 15 mints in a place with no light with 5 µl of Annexin V-FITC and propidium iodide (PI). All procedure was conducted using the Annexin V-FITC/PI Apoptosis Detection Kit (Sigma-Aldrich, U.S.A). based on the manufacturer’s instruction, early apoptosis cells only bind to Annexin-V; however, late apoptosis bind to both Annexin-V and PI and necrotic just bind PI, so make them possible to be distinguished [40, 41]. To do so, BD FACS Calibur flow cytometer (USA) and FlowJo software were used.

### Statistical analysis

The statistical analysis of the data was conducted by GraphPad Prism V9 software, and a two-way ANOVA test was employed to investigate the significance of differences between studied groups. *P*-value less than 0.05 was deemed significant.

## Results

### Characterization of NP-Nrf2_siRNA-CP

NP-Nrf2_siRNA-CP had an average span of 147.9 ± 3.8 nm and a PDI of approximately 0.29 with a surface charge of 14.3 ± 1.8 mV. In addition, CP loading capacity was 11.6%, CP encapsulation efficiency was 75.67%, RTX encapsulation efficiency 76.89%, and siRNA encapsulation efficiency was 90% (Table 1).

**Table 1:** Physicochemical properties of produced nanoparticles

Parameter	CL	CL-RTX	CL-RTX-CP	CL-RTX-CP/ siRNA
**Size (nm)**	90 ± 1.3	100 ± 4.1	135 ± 1.8	147.9 ± 3.8
**PDI**	0.25 ± 0.04	0.3 ± 0.04	0.4 ± 0.03	0.29 ± 0.06
**Zeta potential (mV)**	12.7 ± 2.1	13.5 ± 1.3	12.1 ± 1.3	14.3 ± 1.8
**RTX encapsulation efficiency (%)**		76.89%		
**CP encapsulation efficiency (%)**			75.67%	
**siRNA encapsulation efficiency (%)**				90%
**CP loading capacity (%)**			11.6%	

Based on SEM analysis, NP-Nrf2_siRNA-CP had more roundish-like morphology (Fig. 2b). RTX and CL bioconjugation was confirmed using FTIR spectroscopy data. The spectra of RTX-CL-Nrf2_siRNA-CP showed an absorption band around 1048 cm^−1^ due to new amide bonds which resulted from the interaction between carboxyl groups of RTX and primary amine groups of CL. Moreover, the band at about 1590 cm^−1^ was higher for RTX conjugated NPs compared to native CL due to a larger number of amides II on bioconjugates. Furthermore, the bands inside 3200–3500 cm^−1^ assigned to N–H and O–H vibrations in CL, became narrower for RTX conjugated CL NPs due to reduction in the number of primary amines of CL (Fig. 2c_a-b_).

During stability evaluation, gel electrophoresis study of NP-Nrf2_siRNA-CP after incubation in FBS revealed that siRNA release begins after at least 24 h and could last for up to 48 h (Fig. 2d). Furthermore, comparison of CP and siRNA release pattern in PBS with different PHs (6 and 7.2) showed that the release rate of both CP and siRNA from NP-Nrf2_siRNA-CP was higher in more acidic microenvironment (Fig. 2e).

### NP-Nrf2_siRNA-CP effectively entered leukemic cells and suppressed Nrf2 expression

**Fig. 3:**
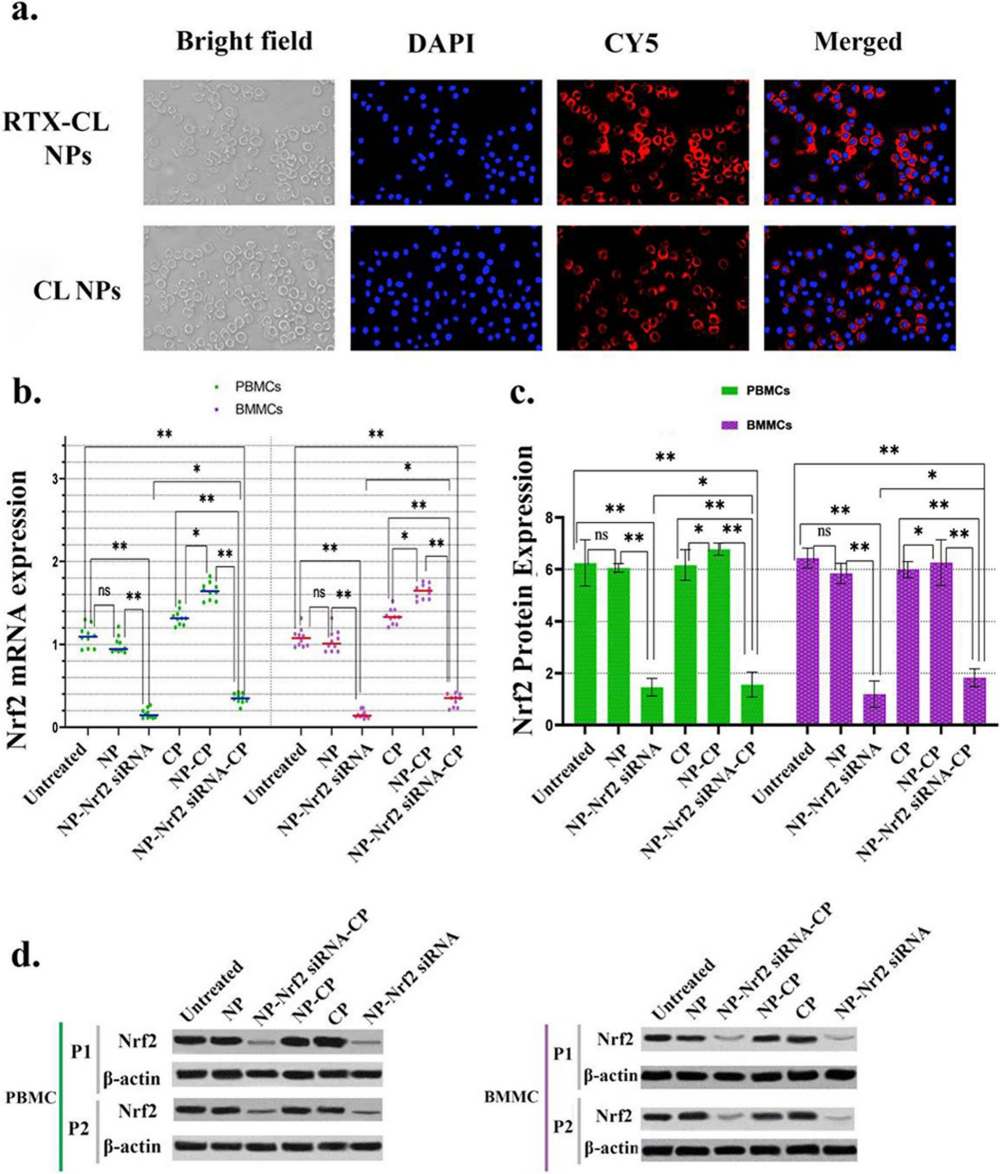
NP-Nrf2_siRNA-CPs entrance efficiency to PBMCs and BMMCs and suppression of Nrf2 mRNA and protein. The simultaneous study of DAPI and CY5 images using confocal laser scanning microscopy (CLSM) show high entrance efficiency of NP-Nrf2_siRNA-CPs to both malignant CLL cells (**a**). Comparison of Nrf2 mRNA (Horizontal line bars represent the mean in each treatment group, and mean of PBMCs = 0.928 and mean of BMMCs = 0.929) (**b**) and protein (Horizontal line bars represent the mean in each treatment group, and mean of PBMCs = 4.712 and mean of BMMCs = 4.589, error bars above and below show the mean with SD) (**c**) expression in both PBMCs and BMMCs respectively based on RT-PCR and western blotting between various therapies. The values were normalized to the level of β-actin mRNA expression. The relative levels of targeted genes mRNA were calculated using the ΔΔCT technique, using the ratio to the value of the untreated cell as a calibrator (**b** and **c**). **d** Shows representative photos of Western blot analysis on PBMCs and BMMCs of CLL patients number 7 (P1) and 3 (P2) after being treated with various agents. The values were normalized to the level of β-actin protein expression, and relative targeted gene protein levels were calculated using the ratio to the value of the untreated cell as a calibrator. Individual patient studies (*n* = 10) show data from primary CLL patient PBMCs and BMMCs samples. *P*-values < 0.05 (*), *P*-values < 0.01 (**), *P*-values < 0.001 (***) and *P*-values < 0.0001 (****)

As explained earlier, a particular form of NP-Nrf2_siRNA-CP conjugated with Cyanine-5 (CY5) dye (NP-CP-Nrf2_siRNA-CY5) was used to examine the cellular uptake. Simultaneous measurement of the intracellular CY5 intensity and nucleus staining with DAPI (4′,6-diamidino-2-phenylindole) demonstrated that conjugating CL with RTX significantly increases the penetration potential of the NPs into leukemic cells (Fig. 3a), which is the key primary step to suppress Nrf2 gene expression by NP-CP-Nrf2_siRNA.In vitro qPCR studies on both PBMCs and BMMCs treated with NP-Nrf2_siRNA-CP (carrying 5 µg anti-Nrf2 siRNA) for 48 h demonstrated a significant decrease in Nrft2 mRNA expression which is corresponding to the high Nrf2 gene silencing efficacy by NP-Nrf2_siRNA-CP (Fig. 3b). Furthermore, applying Western blot analysis on CLL cells (PBMC and BMMC) after 72 h of incubation with NP-Nrf2_siRNA-CP demonstrated a drastic drop in Nrf2 protein expression, similar to Nrf2 gene suppression (Fig. 3c and d).

Surprisingly, contrary to our expectation that Nrf2 expression would be suppressed following CP application, the expression of both Nrf2 protein and mRNA was not notably changed in PBMCs and BMMCs treated with free CP or CP loaded on NPs compared to the control cells (Fig. 3b and c). The overall findings imply that accompanying CP with RTX and anti-Nrf2 siRNA not only augments drug entry to the primary malignant cells in CLL but it also can increase the potential efficacy of CP on these cells by impeding Nrf2 expression.

### Nrf2 inhibition promotes sensitivity of CD20^+^ CLL cells to CP

**Fig. 4:**
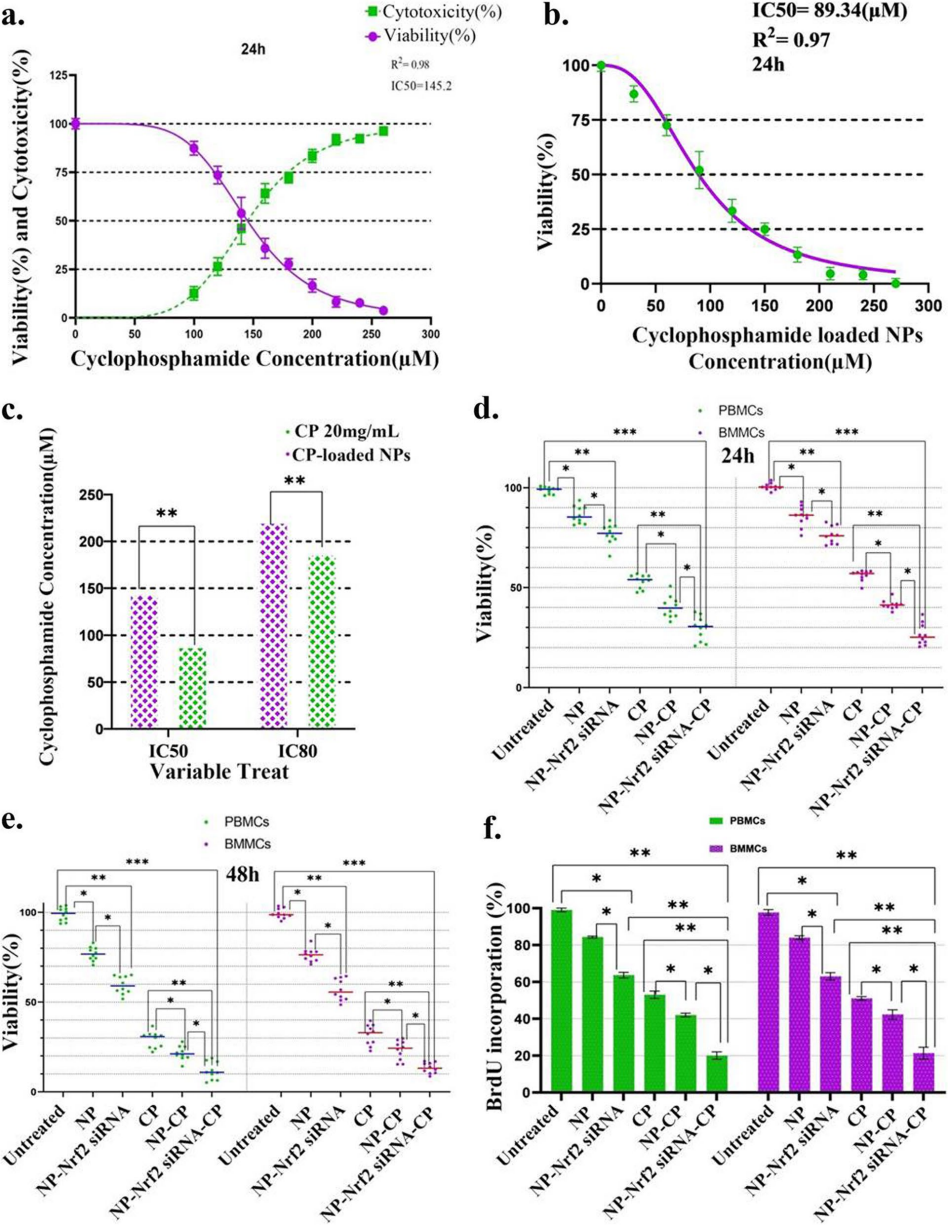
Cell viability assay with MTT. Evaluation of IC50 of CP (**a**), IC50 of CP loaded NPs (**b**), and comparison of IC50 and IC80 of bare CP and CP loaded NPs (**c**). Cell viability assay measurements in malignant PBMCs and BMMCs after both 24 (Horizontal line bars represent the mean in each treatment group, and mean of PBMCs = 64.08 and mean of BMMCs = 64.36) (**d**) and 48 h (Horizontal line bars represent the mean in each treatment group, and mean of PBMCs = 49.57 and mean of BMMCs = 50.10) (**e**) of treatment with different combination therapies is also shown. BrdU incorporation was used to assess the effect of anti-Nrf2 siRNA and CP codelivery on cancer cell proliferation (Horizontal line bars represent the mean in each treatment group, and mean of PBMCs = 60.33 and mean of BMMCs = 59.89, error bars above and below show the mean with SD) (**f**). The data corresponding to the primary PBMCs and BMMCs samples obtained from CLL patients are demonstrated as means of individual patient experiments (*n* = 10). *P*-values < 0.05 (*), *P*-values < 0.01 (**), *P*-values < 0.001 (***) and *P*-values < 0.0001 (****)

MTT assay test was utilized to specify the half-maximal inhibitory concentration (IC50) and 80% inhibitory concentration (IC80) of free CP and NP-Nrf2_siRNA-CP after 24-h ex vivo exposure of both PBMCs and BMMCs. The IC50 and IC80 were considered as minimum concentration of drug needed to cause apoptosis in respectively 50% and 80% of targeted cells. NP-Nrf2_siRNA-CP had both significant lower IC50 (89.34 vs 145.2 μM) and IC80 (188 vs 222 μM) compared to free CP (Fig. 4a-c).

### Nrf2 inhibition augments CP-induced proliferation suppression of CLL cells

As measured by BrdU incorporation assays, all therapeutic agents used to treat CLL cells (PBMC and BMMC) in this study had a more or less suppressive effect on CLL cells proliferation; however, the effect of basic form of our NPs which contained RTX was not statistically significant. Delivering CP using NPs was an efficient way of increasing suppressive effect of this dug on cell proliferation. The combination with NP-Nrf2_siRNA-CP had the most drastic effect on cell proliferation indicating the synergistic roll of anti-Nrf2 siRNA and CP (Fig. 4f).

### Nrf2 inhibition enhances CP-induced cell death in CLL cells

Not even CLL cells (PBMC and BMMC) exposure to NP-Nrf2_siRNA-CP suppressed cell proliferation more significantly than free CP, but it also drastically enhanced CP-induced PCD or apoptosis in CLL cells based on MTT assay study. The findings were valid after both 24 and 48 h of exposure. CLL cells treatment with NP-CP revealed that part of the higher effect of NP-Nrf2_siRNA-CP compared to free CP was due to NP- mediated delivery of CP. Furthermore, RTX containing NPs and NP-Nrf2_siRNA both had significant reductive effect on cell viability. The only therapies capable of apoptosis induction in at least 50% of cells within first 24 h were NP-CP and NP-Nrf2_siRNA-CP. However, after the second 24 h the free CP was also able to reduce cell viability to less than 50% (Fig. 4d and e).

**Fig. 5:**
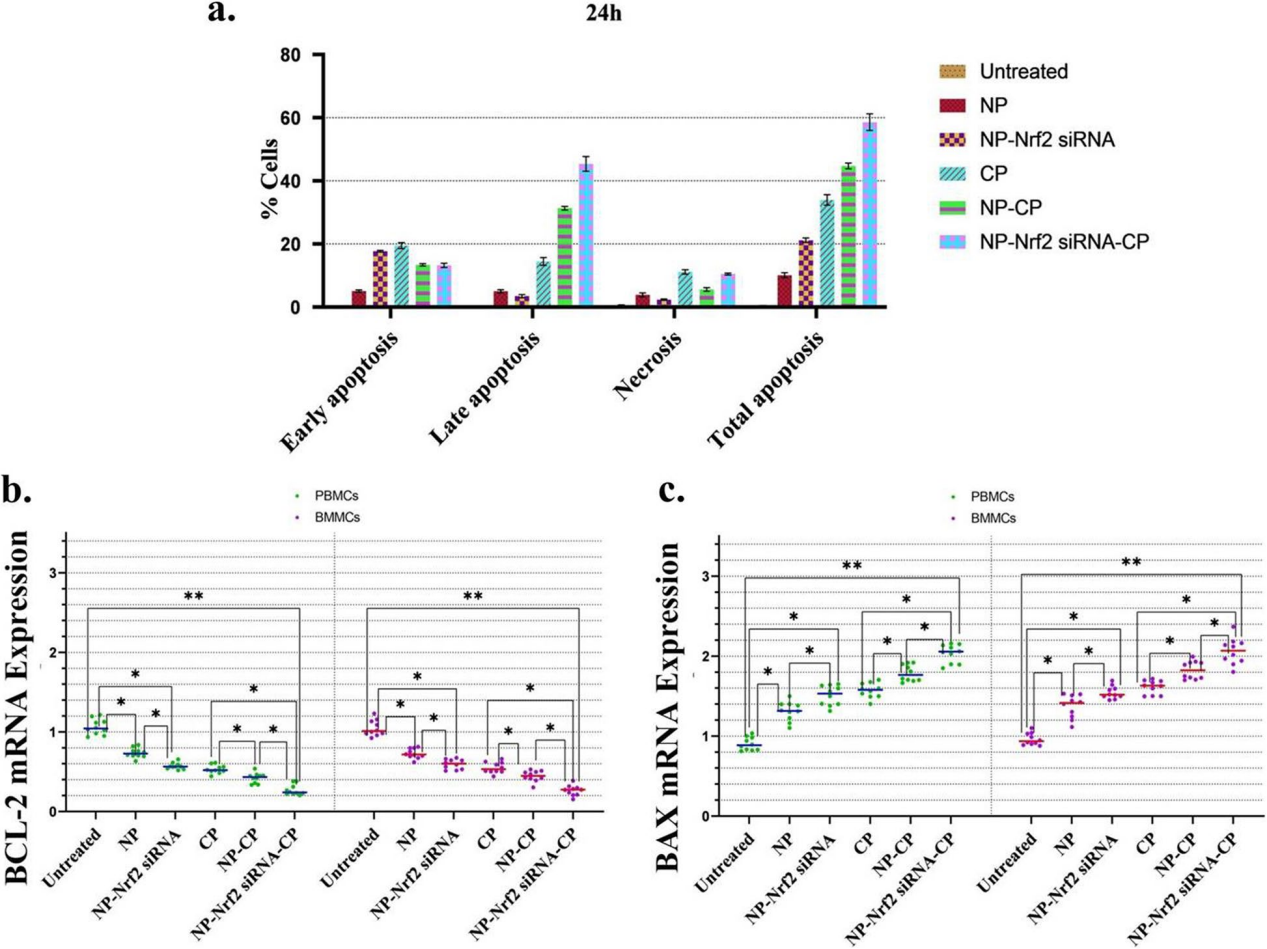
Cell death evaluation after codelivery of anti-Nrf2 siRNA and CP. The proportion of early and late apoptotis and necrosis among CLL patients-derived PBMCs and BMMCs based on Annexin V-FITC and Propidium iodide apoptosis assay(Horizontal line bars represent the mean in each treatment group, and error bars above and below show the mean with SD) (**a**). mRNA expression level of Bcl-2 (Horizontal line bars represent the mean in each treatment group, and mean of PBMCs = 0.59 and mean of BMMCs = 0.60) (**b**) and Bax (Horizontal line bars represent the mean in each treatment group, and mean of PBMCs = 1.52 and mean of BMMCs = 1.56) (**c**) genes based on RT-PCR technique after combination therapy of both PBMCs and BMMCs with various agents are also shown. The values are normalized to the level of β-actin mRNA expression then relative targeted genes mRNA levels determined by taking the ratio to the untreated cell's value as a calibrator based on the ΔΔCT method. The data corresponding to the primary PBMCs and BMMCs samples obtained from CLL patients are demonstrated as means of individual patient experiments (*n* = 10). *P*-values < 0.05 (*), *P*-values < 0.01 (**), *P*-values < 0.001 (***) and *P*-values < 0.0001 (****)

After cells exposure to different therapeutic agents for 24 h, Annexin V-FITC/PI Apoptosis Detection Kit and flow cytometry were utilized to differentiate dead cells with different stages (Fig. 5a). The overall apoptosis and necrosis induction potential of basic NPs was not remarkable. Comparing early and late cell apoptosis stages indicates that apparently, delivering CP using NP-Nrf2_siRNA, and to a lesser extent NPs accelerates apoptosis induction. Despite these differences in cell death stages, the overall 24-h cell apoptosis induction potential of NP-Nrf2-siRNA-CP was greatest among other therapeutic agents, followed by NP-CP and free CP, respectively. The most potent therapy to induce cell necrosis was free CP followed by NP-Nrf2_siRNA-CP. Supplementary Fig. S1 demonstrates more data on the NP/drug-induced apoptotic profile of CLL cells using flow cytometry dot plots.

To further expand the understating of PCD-related effect of combination therapy on CLL cells, the expression level of two PCD regulating genes, Bax (apoptotic) and Bcl-2 (anti-apoptotic), were evaluated. Similar to cell viability-related findings based on the MTT assay study, a meaningful drop in Bcl-2 mRNA expression and a considerable elevation in Bax mRNA expression was detected after all therapeutic agents, with the NP-Nrf2_siRNA-CP being the most potent followed by NP-CP and free CP, respectively (Fig. 5b and c).

## Discussion

B-cell tumor therapeutic interventions usually involve high dose chemotherapy plus monoclonal antibodies (mAbs) as immunotherapy [42, 43]. In spite of favorable survival rates, these regimens are highly toxic for human body and a considerable percentage of patients dealing with leukemia are resistant to them [44]. Therefore, the need for newly developed treatments are inevitable. Multiple underlying mechanism are involved in patient’ resistance against therapy and relapse episodes in CLL, in most of which complex signaling pathways are involved [45, 46]. For instance, genetic alterations in specific oncogenes or oncosuppressors have been linked to failed chemotherapy regimens [47]. To overcome these drawbacks, multiple attempts have been conducted, most of which have led to failure [48]. However, in recent years, nanotechnology-based therapeutic strategies have piqued oncologists' interest due to their potential to provide a new paradigm to overcome challenging curative issues in CLL patients [9, 49]. Actually, nanosized polymeric carriers with few adverse effects have been used to deliver drugs with controlled delivery functions to the human cells for a long time [50, 51]. Therefore, at least theoretically, nanocarriers may have the potential to deliver adequate amount of drug to malignant CLL cells and overcome some overactivated multidrug resistance mechanisms [52].

Here, in this study, we successfully produced RTX-containing NPs loaded with CP and anti-Nrf2 siRNA (NP-Nrf2_siRNA-CP) and targeted CLL cells with them for the first time. we used Chitosan-based NPs which have long been thought to be a suitable therapeutic carrier for targeting malignant cells [53]. The NP-Nrf2_siRNA-CPs used in our study were capable of efficiently entering primary circulating cells acquired from CLL patients and destroying them, in vitro. It’s been previously reported that loading chemotherapeutic agents inside chitosan nanocarriers improves their therapeutic potential and reduces adverse effects in leukemia patients [54]. Furthermore, NPs conjugation with RTX, the FDA-approved anti-CD20 monoclonal antibody for treating CLL [55], promotes cellular absorption and increases tumor-specific drug delivery to leukemic cells [56–58]. Similarly, our newly designed NP-Nrf2_siRNA-CPs were also capable of CLL cells penetration and drug delivery to them.

It’s already known that not all humans are the same in terms of CD20 expression on their B cells and the findings from our patients were confirming of that (Table S1). This indicates the fact that not all CLL cells may be susceptible to RTX conjugated NPs [59], which potentially limits the clinical efficacy of the NP-Nrf2_siRNA-CPs generated herein. However, numerous studies have demonstrated CD20 to have a strong predictive value in CLL patients [60, 61]. In the study by Fang et al., the median expression percentage of CD20 among the 172 studied CLL patients was 97.82%, and patients with positive CD20 B cells had much better prognosis than the others [60]. Speaking of study limitation, although NP-Nrf2_siRNA-CPs had high targeting power and fulfilled their duty against malignant cells, the ex vivo nature of the study doesn’t give much information about the drug function in the CD20-negative in vivo systems; therefore, its use might be limited only to CD20^+^ patients. 

Our study is not the first study to use RTX-coated NPs to target malignant cells [62, 63]. In vitro findings by Mezzaroba et al*.* have demonstrated that anti-CD20-conjugated NPs loaded with hydroxychloroquine and chlorambucil (BNP2) are more efficient than free cytotoxic drugs or RTX at destroying tumor B cells [63]. Moreover, They employed BJAB cell line to create a mouse model of Burkitt Lymphoma (BL) and used them for in vivo studies which showed safe toxicological characteristics and efficacy of BNP2 while treating these mice [63]. Furthermore, not only RTX loaded on NPs enhances the efficacy of chemotherapeutic regimens, but also prescription of free RTX beside cytotoxic agents augments their efficacy. The survey by Chow et al. assessed the effect of RTX monoclonal antibodies on DOHH-2, WSU-NHL, Raji lymphoma cell lines, and peripheral blood samples acquired from CLL patients (*n* = 17). Even though the mere RTX only caused an inconsiderable elevation in apoptosis, it was found that combining it with different cytotoxic drugs significantly reduces the amount of chemotherapeutic agents needed to cause apoptosis, independent of cell saturation with CD20 molecules [62]. As well, many other similar studies have demonstrated efficacy of combination therapy with RTX and chemotherapeutic agents [64–66]. Although conjugating NPs with RTX significantly reduces the potency of complement-dependent cell-mediated cytotoxicity (CDCC), or antibody-dependent cell-mediated cytotoxicity (ADCC), and complement-dependent lysis (CDC), the RTX-induced lethality is still maintained and sufficient through direct inhibition of cell growth signaling responses and apoptosis induction (PCD) [67].

Our novel NPs containing CP as the cytotoxic agent were capable of reducing cell proliferation and apoptosis induction in malignant CLL cells, supported by the alterations in Bcl-2-associated X protein (Bax)(apoptotic) and Bcl-2 (anti-apoptotic) factors in favor of increased apoptosis. Although multiple combination therapies for CLL were developed in recent years and some of them were linked with fewer adverse effects such as Bendamustine plus RTX, the standard primary chemotherapy regimen in CLL patients without del(17p) mutation who are aged between 33 and 81 years old still remains to be the combination of Fludarabine, CP, and RTX [68].

Beside suppression of cell proliferation and inducing cell apoptosis, exploiting anti-nrf2 siRNAs conjugated NPs significantly enhanced CLL cell sensitivity to damage, based on the reduced IC50 and IC80. There were several convincing reasons that Nrf2 inhibition is a favorable goal to increase the sensitivity of CLL cells to CP. First, Nrf2 has a prominent role in sustaining redox homeostasis by adjusting the expression of cytoprotective and oxidation-countering genes [69]. The Nrf2 signaling pathway controls about 600 genes which encode over 200 cytoprotective proteins connected with cell death, cell proliferation, inflammation, malignancy, and drug resistance [11, 69, 70]. Second, it’s been shown that malignant cells such as PBMCs of CLL patients have higher expression of Nrf2 than in healthy individuals [71]. Moreover, it was demonstrated by Sanchez-Lopez et al*.* that promoted expression of Nrf2 in CLL cells mitigates ROS and incudes cell resistance against chemotherapy through activation of mTORC1 and NF-κB pathways corresponding to the p65 phosphorylation which generally leads to the cell survival [11]. Therefore, Nrf2 has been thought of as an attractive target to aim malignant cells by many researchers [24, 25]. Luteolin, Tretinoin, and Brusatol are examples of anti-Nrf2 agents used by various studies to augment CLL cells’ sensitivity to cytotoxic drugs including doxorubicin, arsenic trioxide, and etoposide respectively [72–74].

Despite promising evidence in favor of cytoprotective effects of Nrf2, some conflicting studies believe that Nrf2 activation leads to cytotoxicity and tumor suppression. Bonay et al*.* discovered that activating Nrf2 in THP1 cells with sulforaphane relieves mycobacterial infection and promotes PCD in these cells via caspase 3/7 independent pathways [75]. An additional study by Wu et al*.* reported a drastic increase in apoptosis of TRAMP-C1 cell line and prostatic malignant cells of Transgenic Adenocarcinoma of the Mouse Prostate (TRAMP mice) after treatment with 3,3'-diindolylmethane (DIM) which was accompanied by increased Nrf2 and NQO1 expression, the latter being a Nrf2-target gene. Reduction of DNMT expression followed by suppression of Nrf2 CpG methylation was responsible for elevated Nrf2 expression in this study [76]. Therefore, it seems that Nrf2 has diverse functions according to the type and stage of the cancer. Furthermore, the simultaneous effect of Nrf2 suppression in both healthy and malignant cells in terms of cell proliferation and survival makes targeting Nrf2 a double-edged blade. As a result, Nrf2 has the potential to be classified as both anti/proto-oncogene and further studies are essential to find out benefits of Nrf2 targeting in malignancies.

## Conclusion

Overall, according to the increased expression of Nrf2 in leukemia cells, it can be concluded that mere or combined inhibition of Nrf2 signaling pathways with other immuno/chemotherapeutic agents such as RTX and CP can be a new and potential therapeutic strategy and our newly designed NPs can be an optimal way of drug delivery with these purposes. However, much more research is essential to pave the way for this class of drugs in treating leukemia patients and establish their efficacy and safety.

## Supplementary Information

**Additional file 1:** **Supplementary Table S1.** [77] Patients' characteristics.

**Additional file 2:** **Table S2.** Primer sequences.

**Additional file 3:** **Supplementary Figure S1.** Flow cytometry plots showing apoptosis in response to combination of various treatments.

## Data Availability

The datasets used and/or analysed during the current study are available from the corresponding author on reasonable request.

## Abbreviations

ADCC: Antibody-dependent cell-mediated cytotoxicity
AML: Acute myeloid leukemia
ALL: Acute lymphoblastic leukemia
APL: Acute promyelocytic leukemia
APML: Acute promyelocytic leukaemia
ARE: Antioxidant response element
Bax: Bcl-2-associated X protein
BcL-2: B-cell lymphoma 2
BMNCs: Bone marrow mononuclear cells
BrdU: Bromodeoxyuridine
CDC: Complement-dependent lysis
CDCC: Cell-mediated cytotoxicity
CL: Chitosan lactate
CLL: Chronic lymphocytic leukemia
CML: Chronic myeloid leukemia
CP: Cyclophosphamide
CY5: Cyanine-5
DAPI: 4′,6-Diamidino-2-phenylindole
FBS: Fetal bovine serum
Keap1: Kelch like ECH associated protein 1
mAbs: Monoclonal antibodies
MAPK/ERK: Mitogen-activated protein kinase/ extracellular signal-regulated kinase
Mcl-1: Myeloid leukemia cell differentiation protein1
mTOR: Mammalian target of rapamycin
MTT: (3-(4, 5-Dimethylthiazol-2-yl)-2, 5-diphenyl tetrazolium bromide)
NFκB: Nuclear factor kappa-light-chain-enhancer of activated B cells
NOTCH1: Neurogenic locus notch homolog protein 1
NPs: Nanoparticles
NQO1: NAD(P)H Quinone Dehydrogenase 1
Nrf2: Nuclear factor erythroid 2-related factor 2
PBMCs: Peripheral blood mononuclear cells
PBS: Phosphate buffered saline
PCD: Programmed cell death
PI3K/AKT: Phosphatidylinositol-3-kinase/ Protein kinase B (PKB), also known as Akt
ROS: Reactive oxygen species
RPMI-1640: Roswell Park Memorial Institute medium
RTX: Rituximab
SEM: Scanning electron microscopy
siRNA: Small interfering RNA
TRAMP mice: Transgenic Adenocarcinoma of the Mouse Prostate
