# Supplementary material for: Targeted Silencing of NRF2 by rituximab-conjugated nanoparticles increases the sensitivity of chronic lymphoblastic leukemia cells to Cyclophosphamide
Source: Cell Commun Signal. 2023 Aug 1;21:188. doi: 10.1186/s12964-023-01213-1 (PMC10391779; doi:10.1186/s12964-023-01213-1)
Supplement: Supplementary file 2 — Additional file 1: Supplementary Table S1. [77] Patients' characteristics. [file 12964_2023_1213_MOESM1_ESM.docx]

**Table 1**

| Case No. | CLL  #1 | CLL  #2 | CLL  #3 | CLL  #4 | CLL  #5 | CLL  #6 | CLL  #7 | CLL  #8 | CLL  #9 | CLL #10 | CLL #11 |
| --- | --- | --- | --- | --- | --- | --- | --- | --- | --- | --- | --- |
| Type of sample | PB/BM | PB/BM | PB/BM | PB/BM | PB/BM | PB/BM | PB/BM | PB/BM | PB/BM | PB/BM | PB/BM |
| Subtype | mature B lymphoid cell phenotype | mature B lymphoid cell phenotype | mature B lymphoid cell phenotype | mature B lymphoid cell phenotype | mature B lymphoid cell phenotype | mature B lymphoid cell phenotype | mature B lymphoid cell phenotype | mature B lymphoid cell phenotype | mature B lymphoid cell phenotype | mature B lymphoid cell phenotype | mature B lymphoid cell phenotype |
| Age | 65 | 54 | 74 | 65 | 81 | 54 | 65 | 55 | 66 | 79 | 80 |
| Sex | M | M | M | F | M | M | M | F | F | M | M |
| WBC | 24.27  H | 26.03  H | 45.01  H | 26.83  H | 34.45  H | 20.19  H | 14.21  H | 11.15  H | 23.33  H | 22.02  H | 26  H |
| RBC | 2.41  L | 0.40  L | 3.88  L | 4.08 | 4.72 | 4.96 | 5.34 | 5.49 | 5.022 | 1.03  L | 0.43  L |
| Platelet count | 24  L | 16  L | 326 | 200 | 452  H | 133 | 140 | 269 | 340 | 62  L | 16  L |
| Haemoglobin level | 7.5  L | 3  L | 12.3 | 11.9  L | 12.8 | 14.1 | 14.7 | 13.4 | 14.9 | 3.5  L | 1.1  L |
| HCT | 23.7%  L | 4.4%  L | 35.4%  L | 40.3% | 44.2% | 46.4% | 47% | 45.3% | 45.1% | 11.2%  L | 8.9%  L |
| MCV | 98.1 | 110.2  H | 91.3 | 85.3 | 93.7 | 93.7 | 88.1 | 82.2 | 86.4 | 109.6  H | 113.4  H |
| MCH | 31.1 | 28.5 | 31.7  H | 25.1  L | 27.1 | 28.4 | 27.5 | 24.4  L | 28.5 | 34.3  H | 23.8  L |
| MCHC | 31.7 | 25.9  L | 34.7 | 29.4  L | 29  L | 30.3  L | 31.2  L | 29.7  L | 33 | 31.3  L | 24.8  L |
| RDW | 23.6%  H | 16.6%  H | 14.1% | 13.4% | 14.9% | 12.6% | 13% | 12.9% | 12.2% | 15%  H | 18% |
| NEUT | 5.8%  L | 7.7%  L | 43%  H | 16.9%  L | 10.9%  L | 26.1%  L | 17.6%  L | 30.5%  L | 28.8%  L | 23%  L | 9%  L |
| LYMP | 83.6%  H | 86.3%  H | 55.9%  H | 74.6%  H | 73.7%  H | 65.3%  H | 78%  H | 62%  H | 63.5%  H | 69%  H | 75.3%  H |
| MONO | 1.3%  L | 0.8%  L | 0.9%  L | 1.5%  L | 0.9%  L | 3%  L | 1.2%  L | 3.2%  L | 2.1%  L | 3.2%  L | 1.4%  L |
| CD5 | 96% | 97% | 50% | 81% | 89% | 94% | 81% | 88% | 87% | 82% | 93% |
| CD19 | 88% | 95% | 68% | 94% | 79% | 65% | 78% | 56% | 63% | 86% | 75% |
| CD20 | 83% | 50% | 70% | 60% | 79% | 71% | 70% | 51% | 54% | 58% | 74% |
| CD23 | 71% | 64% | 33% | 79% | 67% | 69% | 81% | 61% | 61% | 77% | 80% |
| liver span (mm) | 138↑ | 188↑ | 125↑ | 114↑ | 104↑ | 112= | 130↑ | 108= | 114↑ | 122↑ | 188↑ |
| Spleen span  (mm) | 176↑ | 159↑ | 90↑ | 84↑ | 154↑ | 96↑ | 66= | 85↑ | 94= | 108↑ | 135↑ |
| Lymphadenopathy (long-axis diameter)) | 12 | - | 1.5 | 0.5 | - | - | 15 | - | 10 | - | 17 |

**Abbreviation :** WBC → white blood cells; RBC → Red blood count; HCT → Hematocrit; MCV → Mean corpuscular volume; MCH → Mean corpuscular hemoglobin; MCHC → Mean Corpuscular Hemoglobin Concentration; RDW → Red Cell Distribution Width; NEUT → Neutrophils; LYMP → Lymphocyte ; MONO → Monocytes.
