# Supplementary material for: Targeted Silencing of NRF2 by rituximab-conjugated nanoparticles increases the sensitivity of chronic lymphoblastic leukemia cells to Cyclophosphamide
Source: Cell Commun Signal. 2023 Aug 1;21:188. doi: 10.1186/s12964-023-01213-1 (PMC10391779; doi:10.1186/s12964-023-01213-1)
Supplement: Supplementary file 3 — Additional file 2: Table S2. Primer sequences. [file 12964_2023_1213_MOESM2_ESM.docx]

| Table 2. PRIMER SEQUENCES | | |
| --- | --- | --- |
| NRF2 | Forward | 5'- GCT GCC TCT GAA GAA GGA GA -3' |
|  | Reverse | 5'- ACA TAC CAC TGT GAG GGC AA -3' |
| BCL-2 | Forward | 5'- TCG CCC TGT GGA TGA CTG A -3' |
|  | Reverse | 5'- CAG AGA CAG CCA GGA GAA ATC A -3' |
| BAX | Forward | 5'- CCC GAG AGG TCT TTT TCC -3' |
|  | Reverse | 5'- GCC TTG AGC ACC AGT TTG -3' |
| β ACTIN | Forward | 5'- CAC CAT TGG CAA TGA GCG GTT C 3' |
|  | Reverse | 5'- AGG TCT TTG CGG ATG TCC ACG T -3' |
