# Supplementary figures and images for: Targeted Silencing of NRF2 by rituximab-conjugated nanoparticles increases the sensitivity of chronic lymphoblastic leukemia cells to Cyclophosphamide
Source: Cell Commun Signal. 2023 Aug 1;21:188. doi: 10.1186/s12964-023-01213-1 (PMC10391779; doi:10.1186/s12964-023-01213-1)

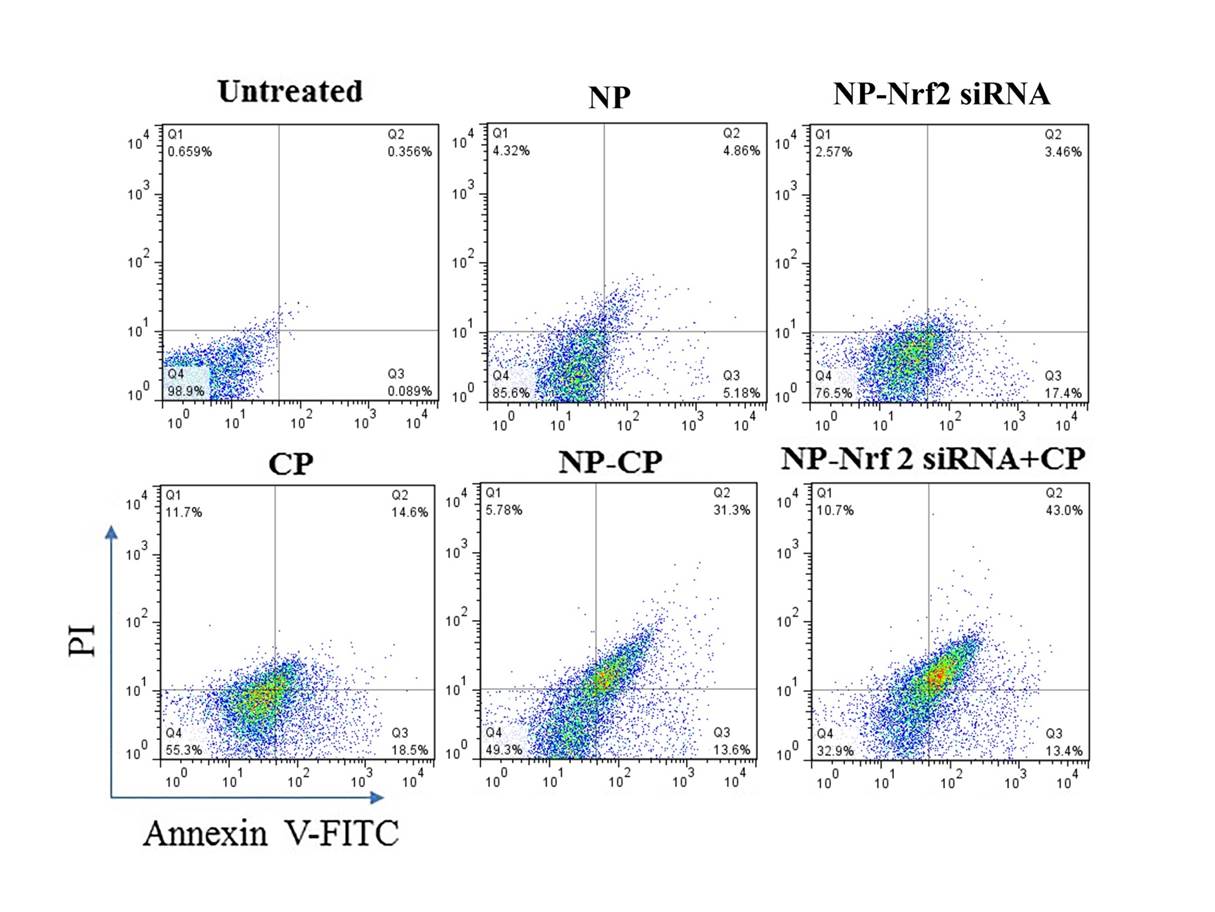

Supplement: Supplementary file 4 — Additional file 3: Supplementary Figure S1. Flow cytometry plots showing apoptosis in response to combination of various treatments. [file 12964_2023_1213_MOESM3_ESM.jpg]
